# Antibiotics and antiseptics for preventing infection in people receiving revision total hip and knee prostheses: a systematic review of randomized controlled trials

**DOI:** 10.1186/s12879-016-2063-4

**Published:** 2016-12-12

**Authors:** Jeffrey Voigt, Michael Mosier, Rabih Darouiche

**Affiliations:** 1 99 Glenwood Rd, Ridgewood, NJ 07450 USA; 2 Washburn University, Morgan Hall, Room 250 J, 1700 SW College Ave, Topeka, KS 66621 USA; 3 Michael E. DeBakey Veterans Affairs Medical Center, 2002 Holcombe Blvd # 128, Houston, TX 77030 USA

## Abstract

**Background:**

Infection rates in revision (second and subsequent) major joint arthroplasty continues to be a significant issue with rates 2–3 times those of primary procedures. The effect of antibiotic and antiseptic prophylaxis on outcomes for this type of surgery has not been adequately reviewed.

**Methods:**

A systematic search of the main databases for randomized controlled trials (RCTs) evaluating antibiotics and antiseptics was conducted to evaluate the predetermined endpoints of infection.

**Results:**

There were five (5) RCTs identified that examined the effects of antibiotic and antiseptic prophylaxis on infections after revision total hip arthroplasty [THA] (total of 304 participants) and total knee arthroplasty [TKA] (total of 206 participants). For TKA, preoperative systemic intravenous (IV) antibiotic prophylaxis plus antibiotic cement may be effective in reducing the incidence of infection in revision TKA at 8+ years. These results however should be interpreted with caution due to the significant biases. For revision THA, there is no RCT evidence that antibiotics/antiseptics have any effect on the infection rate.

**Conclusions:**

There is a lack of high quality data demonstrating an effect of antibiotics or antiseptics on infection rates in revision THA/TKA. Considering the rate of infections in revisions is 2-3X that of primary procedures and; there is a consensus recommendation to use similar antibiotic and antiseptic regimens in both primary and revision procedures, there is a need for high quality studies in revision THA/TKA.

**Electronic supplementary material:**

The online version of this article (doi:10.1186/s12879-016-2063-4) contains supplementary material, which is available to authorized users.

## Background

The use of antibiotic and antiseptic prophylaxis for primary total hip and knee arthroplasty (referred to going forward as THA and TKA) has recently been examined in a systematic review and meta-analysis of randomized controlled trials [1]. In this analysis, it was found that preoperative systemic antibiotic prophylaxis is effective at reducing the infection rate in primary THA (vs. placebo) and that there is no high quality RCT evidence antibiotic prophylaxis is effective at reducing the infection rates in primary TKA. Additionally, it was found that the majority of the US studies examining antibiotic prophylaxis were published back in the 1980s and 1990s. As has been noted, the incidence of diabetes and obesity has increased significantly in these types of patients both for primary and revision THA/TKA [2, 3]; with diabetes and obesity being known and statistically significant risk factors for infection [4–7]. To date there has not been any sort of systematic review of RCTs examining the effect of antibiotic and antiseptic prophylaxis solely on revision THA and TKA. There have been systematic reviews and meta-analysis that have examined the effect of antibiotic prophylaxis on primary and revision THA/TKA collectively [without separating them out] [8–10]. However, studies also acknowledge the fact that the peri-prosthetic infection rate is 2–3 times higher in revision THA and TKA than in primary THA/TKA. Additionally, other studies have stated that patients are at a 9–13 times higher risk of infection in a revision TKA/THA procedure than in a primary THA/TKA [11]. Further, the infection rates in revision THA/TKA have also more than doubled from 1.4% in the 1991–1994 timeframe to 3.0% in the 2007–2010 timeframe [3]. Despite the statistically significant higher infection rates seen in revision THA/TKA, surprisingly, the current strong consensus is that perioperative antibiotic prophylaxis should be the same for primary and uninfected revision THA/TKA [12].

The most common organisms for implant infection are *Staphylococcus aureus* (50–65%) and *Staphylococcus epidermis* (25–30%) [13, 14]. However there is also a risk posed by nosocomial (hospital acquired) bacterial infections that are resistant to the antibiotics commonly used prophylactically in implant surgery [15]. These hospital acquired infections include *Clostridium difficile* (*C. diff*) and methicillin-resistant *Staphylococcus aureus* (MRSA). This is especially important in revision procedures as patients undergoing these procedures have an increased risk of developing *C diff* infections due to their more advanced age and length of hospital stay (relative to primary THA/TKA procedures) [3].

In the US, over the 2006–2012 timeframe (most recent data available on HCUP-Net), there has been a 29% increase in the number of primary THA and TKA implant procedures going from 700,000 in 2006 to 910,000 in 2012 [16]. During this same timeframe, there has been a 35% increase in the number of revision THA and TKA implant procedures (complete or partial revision) - going from 75,000 in 2006 to 104,000 in 2012 [16] (See Table 1). This increase in revision procedures in excess of the increase in the number of primary THA and TKA implants is likely due to the prevalence of over 7 million people living with THA and TKAs [17]. A main reason for revision has been due to infection, with approximately 35% of large joint implants (THA/TKA) being revised for this reason [16].

**Table 1:** Total joint implants - primary and revision procedures US 2006-2012

Year	Prim TKA	% change	Prim THA	% change	Rev TKA	% change	Rev THA	% change	infection TJA	% change	Rev THA/TKA procedures	% revision due to infect
2006	481,804		221,639		43,746		31,185		26,659		74,931	35.6%
2007	532,568	10.5%	244,247	10.2%	47,424	8.4%	34,174	9.6%	29,210	9.6%	81,598	35.8%
2008	591,493	11.1%	265,768	8.8%	56,013	18.1%	37,474	9.7%	33,986	16.4%	93,487	36.4%
2009	596,781	0.9%	274,090	3.1%	54,592	-2.5%	36,695	-2.1%	32,306	-4.9%	91,287	35.4%
2010	631,082	5.7%	290,959	6.2%	62,725	14.9%	41,340	12.7%	34,469	6.7%	104,065	33.1%
2011	617,823	-2.1%	293,117	0.7%	67,061	6.9%	45,212	9.4%	37,064	7.5%	112,273	33.0%
2012	610,409	-1.2%	299,590	2.2%	62,710	-6.5%	41,545	-8.1%	36,650	-1.1%	104,255	35.2%
Percent chg 2006 to 2012		26.7%		35.2%		43.4%		33.2%		37.5%		

It is with these issues in mind that a systematic review was undertaken of randomized controlled trials to examine the effect of antibiotics and antiseptics used peri-procedurally during the revision THA/TKA procedure. Specifically, the objective of this systematic review was to determine if the application of, types of, route administered, timing, and dosage(s) of antibiotics and antiseptics affected the outcome of infection rates seen post-procedurally in revision THA and TKA.

## Methods

### Data sources and searches

The following electronic databases were searched:

- The Cochrane Wounds Group Specialized Register (searched 31 March 2015);
- The Cochrane Central Register of Controlled Trials (CENTRAL; 2015, Issue 3);
- The Database of Abstracts of Reviews of Effects (DARE; 2015, Issue 1);
- The NHS Economic Evaluation Database (NHS EED; 2014, Issue 1);
- Ovid MEDLINE (1948 to March Week 15, 2015);
- Ovid MEDLINE (In-Process & Other Non-Indexed Citations, 31 March 2015);
- Ovid EMBASE (1980 to 2015 Week 15);
- EBSCO CINAHL (1982 to 31 March 2015);
- Network Digital Library of Theses and Dissertations (NDLTD)

Contacting corresponding authors of included trials was attempted (where updated contact information existed) in addition to the manufacturers and distributors of antibiotics (linezolid, quinupristin/dalfopristin, daptomycin, tigecycline, telavancin and other antistaphylococcal agents and antiseptics). The US Food and Drug Administration (FDA) briefing documents used in the licensing of antistaphylococcal agents was also searched. Citation lists of papers identified by the above strategies for further reports of eligible studies were also checked. The following journals were also hand searched:

- *Journal of Bone and Joint Surgery (American volume)* (most recent six months up to 4 September 2015; searched on 4 September 2015);
- *Journal of Bone and Joint Surgery (British volume)* (most recent six months up to 4 September 2015; searched on 4 September 2015);
- *Clinical Orthopedics & Related Research* (most recent six months up to 4 September 2015; searched on 4 September 2015) and;
- *Journal of Antimicrobial Agents & Chemotherapy* (most recent six months up to 4 September 2015; searched on 4 September 2015).

Hand searching the journals above was undertaken because of the time lag between their publication and availability on electronic indexes.

In addition, ClinicalTrials.gov on 4 September 2015 were searched to identify any trials in process or recently completed. Google was searched on 4 September 2015 using the search terms: mupirocin, antibiotic, prophylaxis, revision, and orthopedic. The first 8 pages of hits were evaluated.

Search MeSH terms can be found in Additional file 1: Appendix 1. Two review authors screened the titles and abstracts of all studies identified by the search independently. Upon the verbal agreement of both review authors, we obtained full text versions of all studies identified as potentially relevant, and two review authors assessed them independently against the inclusion criteria. Any disagreement(s) between the two review authors were resolved by discussion or adjudicated by a third author.

### Study selection

A systematic review of randomized controlled trials (RCTs) was undertaken that investigated the effect of perioperative antibiotic prophylaxis with or without antiseptics, on outcomes related to surgical site infections (SSIs) during revision THA or TKA replacement. As it relates to definitions used in this analysis, THA/TKA revision procedures are performed for a number of reasons including: mechanical loosening, dislocation, implant failure/breakage, periprosthetic fracture, periprosthetic osteolysis, bearing surface wear, and infection. We did not include revision THA/TKA for infection as the treatment is antibiotics/antiseptics. We were specifically seeking the effect of antibiotic/antiseptic prophylaxis in the future incidence of infection. Additionally, revision THA/TKA procedures are performed to revise all or some of the components that make up the THA/TKA implant. In this analysis we are defining a revision THA as the repeat (second time or more) replacement of: the femoral implant; and/or the acetabular (socket) component and/or; the acetabular liner (composed of different materials but commonly polyethylene). Similarly, revision TKA is defined as the repeat (second time or more) replacement of: the top/upper portion of the tibial bone (tibial component); and/or the tibial insert (composed of polyethylene); and/or the bottom portion of the femoral bone (or femoral condyles termed the femoral component); and/or the patellar components. It also involved the removal of these implants without replacement. Implants that did not meet this definition of THA and TKA were excluded. The years in which antibiotics and antiseptics were first introduced up to the present were considered. All languages were considered. The PRISMA and CONSORT guidelines were followed.

Two review authors screened the titles and abstracts of all studies identified by the search independently. Upon the agreement of both review authors, we obtained full text versions of all studies identified as potentially relevant, and two review authors assessed them independently against the inclusion criteria. Any disagreement(s) between the two review authors were resolved by discussion or adjudicated by a third author. Only full text versions of studies were considered (published or unpublished). Abstracts and conference proceedings were not considered, unless a full length manuscript existed.

### Data extraction and study quality assessment

A data extraction form was developed (See Additional file 1: Appendix 1). One review author extracted the data and a second review author validated the extracted data (performed via written comments and verbally). If a study had more than one publication, all versions were considered in order to maximize data extraction, and the primary publication was identified, along with the secondary references. Where possible, the original investigators were contacted to request the missing data and this was reported on qualitatively.

### Assessment of risk of bias in included studies

Two review authors independently assessed each included study using the Cochrane Collaboration tool for assessing risk of bias [18]. This tool addresses six specific domains, namely sequence generation, allocation concealment, blinding, incomplete outcome data, selective outcome reporting and other issues (e.g. extreme baseline imbalance; see Additional file 1: Appendix 1 for details of the criteria on which judgements were based). Blinding and completeness of outcome data were assessed for each outcome separately. A ‘Risk of bias’ table was completed for each eligible study. Any disagreement(s) amongst the review authors were discussed to achieve a consensus.

Assessment of risk of bias using a ‘Risk of bias’ summary figure was evaluated, which presents all of the judgements in a cross-tabulation of study by entry. This display of internal validity indicates the weight the reader may give the results of each study.

A separate examination of the results was reported according to journal of publication and country to determine whether results differed according to the impact of the journal (high versus low [19]; and country (location bias [20]. We also assessed studies other than RCTs (i.e. quasi-RCTs) using the same criteria. We incorporated the results of the ‘Risk of bias’ assessment into the review through systematic narrative description and commentary about each of the domains, leading to an overall assessment of the risk of bias of the included studies and a judgement about the internal validity of the results.

### Data synthesis and analysis

If trials included multiple intervention groups (e.g. different antibiotics), the goal was to split the shared control group into two or more groups with smaller sample sizes, depending upon the number of interventions, and include two or more comparisons.

Authors of papers that we identified only as abstracts were contacted to determine whether the full paper had been published in a peer-reviewed journal or was available from the author as an unpublished draft.

### Assessment of reporting biases

Each primary outcome was reported separately. Furthermore, an assessment of publication bias (including a review of unpublished studies); location bias (types of journals) and language bias was made. The results of trials were examined as favorable or not, with the assumption that favorable results demonstrated a positive effect of antibiotic prophylaxis in lowering the infection rate and thus were published (versus not published) [21]. Location bias refers to more significant results being published in less-respected/low impact factor journals [19]. Lastly, an analysis was made of the reporting of outcomes (reporting bias) as identified below in the ‘Risk of bias’ tables.

## Results

### Results of the search

**Fig. 1:**
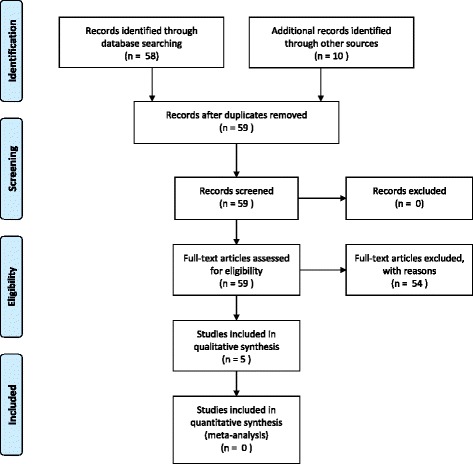
PRISMA flow diagram

The electronic searches identified a total of 58 potentially relevant reports. We obtained abstracts for all 58 for further review and evaluation. (See PRISMA Flow Diagram, Fig. 1 for included and excluded studies).

Bibliographic reference checking of a Health Technology Assessment [9], other systematic review articles [8], and of current concept review articles [20, 21], identified six additional articles [22–27]. Further hand searching of a Cochrane Review [28] (found through the electronic search) identified an additional study [29]. Hand searching of journals for RCTs also identified three articles [30–32]. Thus a total of ten studies were identified through hand searches.

In total 68 study reports were identified.

### Included studies

Five RCTs met the inclusion criteria for the review [33–37]. Two authors were contacted to clarify questions on study design [33] and the breakout of infections by revision THA/TKA [37]. In Chiu [33], both the lead and co-authors never responded to email queries. Phillips [37] responded on 1 September 2015 that he did not have the time to provide additional information (thus a sensitivity analysis was performed on the worst case scenarios to determine if a statistical difference existed). All of the studies except one (Chiu 2009) [33] were conducted in the US. All studies except for one [36] were single center RCTs. Two studies were >20 years old [34, 36]. Table 2 shows a breakout of the studies by: comparison; outcome and total number of patients. In total there were 510 patients.

**Table 2:** Comparisons made in RCTs on the outcome of infection

Number patients				
Study	Comparison	Outcome	Revision THA	Revision THA
Chiu 2009	Antibiotic impregnated bone cement vs. none	Infection >8 year follow-up	183	0
DeBenedictis 1984	1st vs. 2nd generation cephalosporin	Infection 12 months		1
Mauerhan 1994	1st vs. 2nd generation cephalosporin	Infection 12 months	62	132
Jacobson 2005	DuraPrep plus Ioban drape vs. povidone iodine antiseptic	30 day infection	11	9
Phillips 2014	5 day pre-op mupirocin nasal nare application vs. 2 hour pre-op povidone iodine antiseptic	3 month infection	48	64
Total			304	206

For the included studies, the comparisons by type of implant, along with summary data on trial design/methodology, sample size, setting, baseline characteristics of patients, and important differences see the Characteristics of the included studies as found in Additional file 2: Appendix 2.

### Excluded studies

#### Excluded study – characteristics

Six studies were excluded as they were duplicates of other studies. These duplicates were [32, 38–42]. Thirty one studies were excluded as they were randomized trials performed on primary THA and TKA only [22, 25, 26, 27, 31, 43–67]. Nine studies were excluded due to the fact that the number of revision THA/TKA procedures and corresponding infections could not be broken out from primary THA/TKA [29, 68–75]. Three studies were excluded as they were for revisions of infected primary THA/TKA and the antibiotics administered were for treatment and not for prophylaxis [76–78]. Three studies were excluded due to the fact that the procedures were not identified as a THA/THA but as an endoprosthesis [79–81]. One study was excluded due to the fact that in the 4 revision THA procedures identified, it could not be determined to which treatment group the patients were allocated to (flucloxacillin or cephaloridine antibiotic prophylaxis [30]. One study was excluded because the procedures being performed were adult orthopedic surgical cases which excluded total joint arthroplasty patients [82]. Lastly, one study was excluded due to it being a retrospective review of the timing of antibiotic prophylaxis [83]. Thus in total 55 studies were excluded with reasons. For further detail on excluded studies, see the Characteristics of excluded studies as found in Additional file 2: Appendix 2.

### Risk of bias in included studies

#### Generation of the randomization sequence

In the trial with the largest number of patients [33], the risk of bias for randomization was high, considering the use of an “odd-even” allocation. In 3 of the trials, the randomization scheme was clearly identified [35, 36, 37]. In one study, the randomization scheme was unclear [34].

### Allocation concealment

In all of the trials it was unclear as to when allocation to the treatment occurred.

### Blinding (performance bias and detection bias)

In 3 of the trials [33, 35, 37] the clinician performing the procedures and/or the patient were aware of which treatment group they were allocated to. In 4 of the trials [33, 34, 35, 36] it was unclear if the clinician assessing for the outcome of infection was aware of which treatment group the patient was allocated to.

### Incomplete outcome data (attrition bias)

In 3 of the trials there was a high risk of bias related to attrition of patients on follow up [33, 35, 36]).

### Selective reporting (reporting bias)

In 2 of the studies the risk of selective reporting was high. In DeBenedictis [34], adverse drug reactions identified as an outcome in the methods section were not reported on in the results section. In Phillips 2014, infection and adverse events at 12 months was identified as an endpoint in the methods section but only 3 months of follow up on infections and adverse events were reported on in the results section.

### Other potential sources of bias

In 3 of the trials there was a high risk of other bias due to: DeBenedictis 1984 [34] - financial assistance from a manufacturer of one of the antibiotics used; Jacobson 2005 [35] - financial assistance from a manufacturer for the antiseptic used and in the writing of the manuscript; and Phillips 2014 [37] - the manufacturer of one of the products used providing a research grant for the study.

**Fig. 2:**
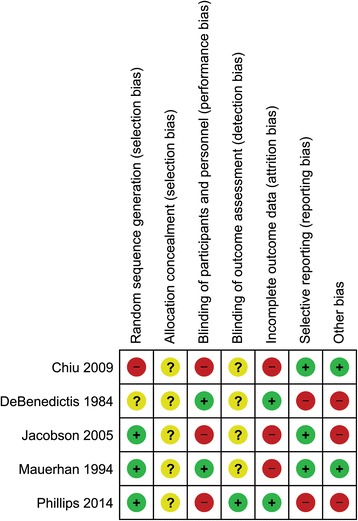
Risk of bias summary

**Fig. 3:**
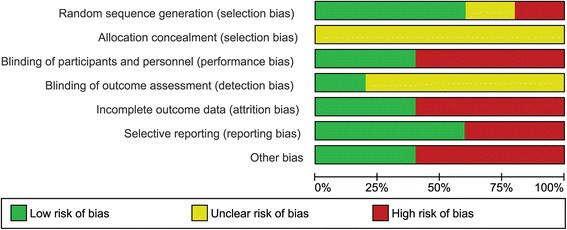
Risk of bias graph

Risk of bias is found in Fig. 2 (Risk of bias summary) and Fig. 3 (risk of bias graph) and; as well Additional file 2: Appendix 2.

### Effects of interventions (main outcome of incidence of infection)

Statistically significant findings (using Fisher exact test) were identified when comparing vancomycin impregnated cement along with IV cefazolin to IV cefazolin in revision TKA procedures [33] (*P* = 0.0129). Non-significant findings on the 1 year outcome of infection were identified in comparing 1st generation vs. 2nd generation cefazolin in revision TKA [36] (RR = 0.29; 95% CI: 0.01 to 6.95; *P* = 0.45). Further as it relates to the use of mupirocin vs. povidone iodine (PI) nasal antisepsis there was no statistically significant difference in the outcome of infection at 30 days for either revision THA or revision TKA. For this particular analysis, since we were unable to obtain the results from the authors of the article [37] we performed a sensitivity analysis on the incidence of infection in either treatment group (mupirocin vs. PI) using the worst case scenarios on the incidence of infections. In all cases, the differences did not reach statistical significance.

## Discussion

Based on the available data and its relative “poor quality” (i.e. studies with significant biases), no recommendations can be made regarding antibiotic/antiseptic prophylaxis regimens for revision THA/TKA. There is a worrisome lack of data demonstrating any effect of prophylactic antibiotic and antiseptic use on the incidence of infection in revision THA/TKA. In total there were 510 revision THA/TKA patients who could be identified in any sort of randomized trial. Additionally, 183 of these 510 patients come from a trial that is considered poorly designed from a randomization standpoint [18]. Considering the infection rate in revision THA and TKA is significantly higher than that in primary THA/TKA [7, 8] and; that the consensus recommendation by the Orthopaedic Research Society [12] is that the preoperative antibiotic regimens should be no different between primary and revision THA/TKA - there appears to be no effective prophylaxis regimen(s) that can reduce the infection rate seen in revision THA/TKA. It should be noted that this consensus recommendation has been made using non-randomized studies [12], which thus may render these conclusions questionable.

In the past, the meta-analytic examination of prophylactic antibiotics and antiseptics for preventing infection in primary and revision THA/TKA had not been broken out from primary and revision THA/TKA procedures. For this particular analysis it meant that 9 studies had to be excluded from the overall analysis due to this [29, 68–75]. Additionally, THA and TKA have also not been broken out on the outcome of infection [1] - THA and TKA have traditionally been combined on the outcome of infection. This is also a concern considering the fact that twice as many people receive primary TKA vs. THA; are 20% heavier and live 25% longer than they did back in the late 1980s–1990s; are more physically active; that diabetes is 4 times as prevalent in the US vs. 25 years ago [84] and; that diabetes along with obesity are risk factors for infection. [4–7]. This likely means at many patients will outlive their primary THA/TKA, will be sicker and require a revision procedure.

## Conclusions

This analysis supports the contention that more studies need to be initiated examining; what if any antibiotic/antiseptic prophylactic regimen(s) are effective at reducing the infection rate in revision THA/TKA. A potentially reasonable place to start, based on the results from Chiu 2009 [33], is with the use of antibiotic impregnated cement plus IV antibiotics in revision TKA and; engaging in a trial that is randomized appropriately with blinding of clinical assessors (both issues with Chiu 2009 [33]). Further there appears to be some clinical benefit in using antibiotic impregnated bone cement in primary THA or TKA for reducing the infection rate based on a recent systematic review and meta-analysis [85] and in a recent systematic review [10]. These analyses however, included non-randomized studies but may indicate a potential effect of antibiotic cement in revision THA/TKA [10]. There are also several studies being undertaken which examine the use of intraoperative local antibiotic administration in revision TKA [86] (NCT02020031); preoperative antisepsis in revision THA/TKA [87] (NCT02469311) and; betadine lavage intraoperative and just prior to surgical closure in revision TKA [88] (NCT01175044). The numbers of patients involved in some of these are likely to be too small to demonstrate any statistically significant effect [86]. There are also no studies examining next generation antibiotics specifically for revision THA/TKA. Additional RCT studies on antibiotic/antiseptic prophylaxis in revision THA/TKA should be easier to undertake as the pool of potential patients to include increases each year (Table 1) and the incidence of infections in revision THA/TKA is 2–3 times that of primary THA/TKA [7, 8].

## Additional files

Additional file 1: Appendix 1. Search terms and data extraction formR2. (DOCX 25 kb)
Additional file 2: Appendix 2. Characteristics of included and excluded studies and ongoing studiesR2. (DOCX 123 kb)

## Abbreviations

C. diff: Clostridium difficile
CENTRAL: Cochrane Central Register of Controlled Trials
CONSORT: Consolidated Standards of Reporting Trials
FDA: Food and Drug Administration
MRSA: Methicillin-resistant *Staphylococcus aureus*
NDLTD: Network Digital Library of Theses and Dissertations
PRISMA: Preferred Reporting Items for Systematic Reviews and Meta-Analyses
RCT: Randomized controlled trial
SSI: Surgical Site Infection
THA: Total hip arthroplasty
TKA: Total knee arthroplasty
