# Supplementary material for: Antibiotics and antiseptics for preventing infection in people receiving revision total hip and knee prostheses: a systematic review of randomized controlled trials
Source: BMC Infect Dis. 2016 Dec 12;16:749. doi: 10.1186/s12879-016-2063-4 (PMC5153681; doi:10.1186/s12879-016-2063-4)
Supplement: Additional file 1: — Appendix 1. Search terms and data extraction formR2. (DOCX 25 kb) [file 12879_2016_2063_MOESM1_ESM.docx]

**Appendix 1: MeSH search terms and data collect form used to collect data:**

CENTRAL on 31 March 2015 was searched using the following exploded MeSH headings and keywords:

#1 MeSH descriptor Vancomycin explode all trees
#2 MeSH descriptor Cephalosporins explode all trees
#3 MeSH descriptor Ciprofloxacin explode all trees
#4 MeSH descriptor Ofloxacin explode all trees
#5 MeSH descriptor Aztreonam explode all trees
#6 MeSH descriptor Trimethoprim-Sulfamethoxazole Combination explode all trees
#7 MeSH descriptor Oxazolidinones explode all trees
#8 (antibiotic* or antibacterial* or antimicrobial* or cefazolin or cefepime or vancomycin or aztreonam or ciprofloxacin or levaquin or trimethoprim or linezolid):ti,ab,kw
#9 MeSH descriptor Anti-Infective Agents, Local explode all trees
#10 antiseptic*:ti,ab,kw
#11 MeSH descriptor Iodophors explode all trees
#12 MeSH descriptor Chlorhexidine explode all trees
#13 MeSH descriptor Povidone-Iodine explode all trees
#14 MeSH descriptor Alcohols explode all trees
#15 (iodophor* or povidone or iodine or chlorhexidine or betadine or alcohol*):ti,ab,kw
#16 (#1 OR #2 OR #3 OR #4 OR #5 OR #6 OR #7 OR #8 OR #9 OR #10 OR #11 OR #12 OR #13 OR #14 OR #15)
#17 MeSH descriptor Surgical Wound Infection explode all trees
#18 MeSH descriptor Surgical Wound Dehiscence explode all trees
#19 (surg* NEAR/5 infect*):ti,ab,kw
#20 (surg* NEAR/5 wound*):ti,ab,kw
#21 (surg* NEAR/5 site*):ti,ab,kw
#22 (surg* NEAR/5 incision*):ti,ab,kw
#23 ((postoperative post-operative or postoperative) NEXT (wound NEXT infection*)):ti,ab,kw
#24 (#17 OR #18 OR #19 OR #20 OR #21 OR #22 OR #23)
#25 MeSH descriptor Replacement, Arthroplasty explode all trees

The above strategy was also used to search Ovid MEDLINE, Ovid EMBASE and EBSCO CINAHL. We also combined the Ovid MEDLINE search with the Cochrane Highly Sensitive Search Strategy for identifying randomized trials in MEDLINE: sensitivity- and precision-maximizing version^^[[1]](#endnote-1)^^. The EMBASE and CINAHL searches were also combined with the trial filters developed by the Scottish Intercollegiate Guidelines Network^^[[2]](#endnote-2)^^. There were no restrictions on the basis of date or language of publication.

**Data extraction form:**

**Name of person/reviewer extracting data:**

**Author of article:**

**Title:**

**Source (e.g. Journal title):**

**Date of study:**

**Study location (geographical):**

**Care setting (e.g. hospital):**

**Inclusion/exclusion criteria (list of patient inclusion and exclusion criteria)**

***Inclusion*:**

***Exclusion*:**

**Sample size:**

*number in each arm of trial*

*a priori power calculation?*                     **YES    NO     NOT STATED**

*trial powered adequately?*

**Patient baseline characteristics:**

- *age range:*
- *gender:*
- *medical condition(s):*

**TRIAL DESIGN DETAILS:**

Single-center/multicenter trial?

**Study type**

*randomized controlled trial/matched control/unmatched concurrent control/historic control:*

**Allocation**

- *was it random?* **YES    NO     NOT STATED**
- *method of randomization:*
- *was it concealed?***YES    NO     NOT STATED**

**Intervention details**

- *care setting:*
- *treatment group(s):*
- *control(s):*

*co-interventions:*

*duration of intervention:*

- *who delivered intervention?*
- *was the provider blinded?* **YES    NO     NOT STATED**
- *was the patient blinded?* **YES    NO     NOT STATED**

**Outcome measures**

- *what were they?*
- *methods of assessing outcome measures:*
- *blind assessment?* **YES    NO     NOT STATED**
- *when were they measured?*
- *validity of assessment:*
- *inter-assessor reliability:*
- *length of follow-up:*

**Costs**

- *considered?***YES    NO     NOT STATED**
- *cost-effectiveness details:*
- *Results:*

**Analysis:**

- *description of analysis employed:*
- *statistical methods:*
- *comparisons made:*
- *intention to treat analysis?*
- *adjustment for confounding?*
- *subgroups considered:*
- *exploration of heterogeneity:*

**Results:**

*Missing data:*

*length of follow-up:*

*withdrawals/drop outs - are proportion and characteristics of participants lost to follow-up comparable for the study groups at the end of the trial?*

*reasons for withdrawal:*

lost to follow-up:

Number of infections (primary outcome):

- *Intervention arm (1):*
- *Intervention (or control) arm (2):*
- *Intervention arm (if more than 2 intervention arms are included in the trial):*
- *Intervention are (if more than 2 intervention arms are included in the trial):*

Number of adverse events:

*Intervention arm (1):*

- *Intervention (or control) arm (2):*
- *Intervention arm (if more than 2 intervention arms are included in the trial):*
- *Intervention are (if more than 2 intervention arms are included in the trial):*

**Conclusions:**

**Implications (e.g. for practice):**

**Other comments:**

*Methodological quality of study:*

*comparability of intervention:*

*baseline comparability:*

**Criteria for assessing potential sources of bias**

**1.  Was the allocation sequence randomly generated?**

**Low risk of bias**

The investigators describe a random component in the sequence generation process such as: referring to a random number table; using a computer random number generator; coin tossing; shuffling cards or envelopes; throwing dice; drawing of lots.

**High risk of bias**

The investigators describe a non-random component in the sequence generation process. Usually, the description would involve some systematic, non-random approach, for example: sequence generated by odd or even date of birth; sequence generated by some rule based on date (or day) of admission; sequence generated by some rule based on hospital or clinic record number.

**Unclear**

Insufficient information about the sequence generation process provided to permit judgement of low or high risk of bias.

**2.  Was the treatment allocation adequately concealed?**

**Low risk of bias**

Participants and investigators enrolling participants could not foresee assignment because one of the following, or an equivalent method, was used to conceal allocation: central allocation (including telephone, web-based and pharmacy-controlled randomization); sequentially numbered drug containers of identical appearance; sequentially numbered, opaque, sealed envelopes.

**High risk of bias**

Participants or investigators enrolling participants could possibly foresee assignments and thus introduce selection bias, such as allocation based on: using an open random allocation schedule (e.g. a list of random numbers); assignment envelopes used without appropriate safeguards (e.g. if envelopes were unsealed or non­opaque or not sequentially numbered); alternation or rotation; date of birth; case record number; any other explicitly unconcealed procedure.

**Unclear**

Insufficient information provided to permit judgement of low or high risk of bias. This is usually the case if the method of concealment is not described or not described in sufficient detail to allow a definite judgement, for example if the use of assignment envelopes is described, but it remains unclear whether envelopes were sequentially numbered, opaque and sealed.

**3.  Blinding - was knowledge of the allocated interventions adequately prevented during the study?**

**Low risk of bias**

Any one of the following.

- No blinding, but the review authors judge that the outcome and the outcome measurement are not likely to be influenced by lack of blinding.
- Blinding of participants and key study personnel ensured, and unlikely that the blinding could have been broken.
- Either participants or some key study personnel were not blinded, but outcome assessment was blinded and the non-blinding of others was unlikely to introduce bias.

**High risk of bias**

Any one of the following.

- No blinding or incomplete blinding, and the outcome or outcome measurement is likely to be influenced by lack of blinding.
- Blinding of key study participants and personnel attempted, but likely that the blinding could have been broken.
- Either participants or some key study personnel were not blinded, and the non-blinding of others was likely to introduce bias.

**Unclear**

Either of the following.

- Insufficient information provided to permit judgement of low or high risk of bias.
- The study did not address this outcome.

**4.  Were incomplete outcome data adequately addressed?**

**Low risk of bias**

Any one of the following.

- No missing outcome data.
- Reasons for missing outcome data unlikely to be related to true outcome (for survival data, censoring unlikely to be introducing bias).
- Missing outcome data balanced in numbers across intervention groups, with similar reasons for missing data across groups.
- For dichotomous outcome data, the proportion of missing outcomes compared with observed event risk not enough to have a clinically relevant impact on the intervention effect estimate.
- For continuous outcome data, plausible effect size (difference in means or standardized difference in means) among missing outcomes not enough to have a clinically relevant impact on observed effect size.
- Missing data have been imputed using appropriate methods.

**High risk of bias**

Any one of the following.

- Reason for missing outcome data likely to be related to true outcome, with either imbalance in numbers or reasons for missing data across intervention groups.
- For dichotomous outcome data, the proportion of missing outcomes compared with observed event risk is enough to induce clinically relevant bias in intervention effect estimate.
- For continuous outcome data, plausible effect size (difference in means or standardized difference in means) among missing outcomes is enough to induce clinically relevant bias in observed effect size.
- ‘As-treated’ analysis done with substantial departure in the intervention received from that assigned at randomization.
- Potentially inappropriate application of simple imputation.

**Unclear**

Either of the following.

- Insufficient reporting of attrition or exclusions to permit judgement of low or high risk of bias (e.g. number randomized not stated, no reasons for missing data provided).
- The study did not address this outcome.

**5.  Are reports of the study free of suggestion of selective outcome reporting?**

**Low risk of bias**

Either of the following

- The study protocol is available and all of the study’s pre-specified (primary and secondary) outcomes that are of interest in the review have been reported in the pre-specified way.
- The study protocol is not available but it is clear that the published reports include all expected outcomes, including those that were pre-specified (convincing text of this nature may be uncommon).

**High risk of bias**

Any one of the following.

- Not all of the study’s pre-specified primary outcomes have been reported.
- One or more primary outcomes are reported using measurements, analysis methods or subsets of the data (e.g. subscales) that were not pre-specified.
- One or more reported primary outcomes were not pre-specified (unless clear justification for their reporting is provided, such as an unexpected adverse effect).
- One or more outcomes of interest in the review are reported incompletely so that they cannot be entered in a meta-analysis.
- The study report fails to include results for a key outcome that would be expected to have been reported for such a study.

**Unclear**

Insufficient information to permit judgement of low or high risk of bias. It is likely that the majority of studies will fall into this category.

**6.  Other sources of potential bias**

**Low risk of bias**

The study appears to be free of other sources of bias.

**High risk of bias**

There is at least one important risk of bias. For example, the study:

- had a potential source of bias related to the specific study design used; or
- had extreme baseline imbalance; or
- has been claimed to have been fraudulent; or
- had some other problem.

**Unclear**

There may be a risk of bias, but there is either:

- insufficient information to assess whether an important risk of bias exists; or
- insufficient rationale or evidence that an identified problem will introduce bias.

1. Lefebvre C, Manheimer E, Glanville J, on behalf of the Cochrane Information Retrieval Methods Group. Chapter 6: Searching for studies. In: Higgins JPT, Green S (editors). Cochrane Handbook for Systematic Reviews of Interventions Version 5.1.0 (updated March 2011). The Cochrane Collaboration, 2011. Available from [www.cochrane-handbook.org](http://www.cochrane-handbook.org). [↑](#endnote-ref-1)
2. Scottish Intercollegiate Guidelines Network (SIGN). Search Filters. <http://www.sign.ac.uk/methodology/filters.html#random> (Accessed 4 December 2014). [↑](#endnote-ref-2)
