# Supplementary material for: Antibiotics and antiseptics for preventing infection in people receiving revision total hip and knee prostheses: a systematic review of randomized controlled trials
Source: BMC Infect Dis. 2016 Dec 12;16:749. doi: 10.1186/s12879-016-2063-4 (PMC5153681; doi:10.1186/s12879-016-2063-4)
Supplement: Additional file 2: — Appendix 2. Characteristics of included and excluded studies and ongoing studiesR2. (DOCX 123 kb) [file 12879_2016_2063_MOESM2_ESM.docx]

**Appendix 3: Characteristics of included and excluded studies**

**Included studies – characteristics and risks of bias**

**Chiu 2009**

| **Methods** | Single center Quasi-RCT. Performed during the 1993-2004 timeframe. Study performed in Taiwan. |
| --- | --- |
| **Participants** | Patients undergoing first time revision total knee arthroplasty. There were 90 patients in Group 1 whose average age was 70±7.8 years (range 48-89). The main reasons for revision were loosening (n=69) and wear (n=21). M/F = 55/35. There were 93 patients in Group 2 whose average age was 71±8.4 years (range 55-90). The main reasons for revision were loosening (n=64) and wear (n=29). M/F = 61/32.  All patients entered into the trial for first time revision were determined preoperatively and intraoperatively to be without infection (i.e. not preoperative symptoms or signs of infection, a normal preoperative erythrocyte sedimentation rate and C-reactive protein level, and negative intraoperative cultures).  Exclusions were: diabetes, peripheral arterial disease, psoriasis, any previous lower-extremity infection, osteomyelitis, malignant tumor, and treatment with immunosuppressive therapy. |
| **Interventions** | Group 1 (n=90): IV bolus cefazolin (500 mg) plus gentamicin (80 mg) before the operation; IV injections of cefazolin (500 mg every 6 hours) and gentamicin (80 mg every 12 hours) were given for 36 hours after the operation and then oral cefazolin (500 mg every 6 hours) was given for another 7 days. Simplex-P cement was used to fix the components during the procedure.  Group 2 (n=93): IV bolus cefazolin (500 mg) plus gentamicin (80 mg) before the operation; IV injections of cefazolin (500 mg every 6 hours) and gentamicin (80 mg every 12 hours) were given for 36 hours after the operation and then oral cefazolin (500 mg every 6 hours) was given for another 7 days. Simplex-P cement impregnated with 1 gm vancomycin was used to fix the components during the procedure. |
| **Outcomes** | Infection up to an average 89 month follow up. Infections were classified as either superficial or deep and according the system for classification of infections describe by Tsukayama et al. In this classification superficial infection is defined by: <4 weeks duration, fever, inflammation, fluid/pus, no sinus, positive culture, and no extension through the capsule. Deep infection is defined by: <4 weeks duration, fever, inflammation, fluid/pus, no sinus, positive culture, and with extension through the capsule |
| **Notes** | Study received IRB approval and all patients provided written informed consent.  No external funding was provided for the study.  There were 6 deep (2 acute postoperative; 4 late chronic) and 1 superficial infection (21 days post revision surgery) identified in Group 1. There were no superficial infection and; no deep infections in Group 2.  No component loosening or other complications developed during the follow up period.  Sent email to Dr. Chiu on 8/26/15 and 9/1/15 - no reply. Sent email to Dr. Lin (second author) on 9/1/15 - no response. Email requests were for blinding (assessor); and allocation concealment. Since these questions were not answered, the judgement for both was considered unclear. |

**Risk of bias table**

| **Bias** | **Authors' judgement** | **Support for judgement** |
| --- | --- | --- |
| Random sequence generation (selection bias) |  | Quasi-RCT with use of odd chart numbers for enrollment in Group 2 (receiving cement impregnated with vancomycin) and even chart numbers for enrollment in Group 1 (receiving cement only) |
| Allocation concealment (selection bias) |  | Not clear as to what point in the pre-procedure/procedure the patients were randomized to each treatment arm. |
| Blinding of participants and personnel (performance bias) |  | Quote: *"Although the patients were blinded to whether or not the cement was impregnated with vancomycin, the surgeon was not."* |
| Blinding of outcome assessment (detection bias) |  | Not clear from the paper as to whether clinical assessors were blinded to the treatment arms. |
| Incomplete outcome data (attrition bias) |  | Patients undergoing repeat revision were excluded (10) and those lost to follow up (6) |
| Selective reporting (reporting bias) |  | Infection as identified in the methods section was reported on in the results section. |
| Other bias |  | No other biases were identified. |

**DeBenedictis 1984**

| **Methods** | Single-center RCT conducted from June 1981-January 1982 |
| --- | --- |
| **Participants** | Patients receiving either a TKA or THA  Group 1 (n = 37): mean age = 66 years; male:female = 15:22  Group 2 (n = 39): mean age = 69 years; male:female =16:23  Exclusion criteria: evidence of joint infection, a history of sensitivity to penicillin or cephalosporins, significant renal impairment, or interfering antimicrobial therapy |
| **Interventions** | Group 1: 1 g cefonicid (2nd generation cephalosporin) administered IM or IV 30 min prior to incision and once daily for 3 days. Placebo infusions of physiologic saline were used to bring the total daily administration up to 3 doses (n: THA = 1)  Group 2: 1 g cefazolin (1st generation cephalosporin) 30 min prior to incision and every 8 h for 72 h post surgery (n: THA = 0; TKA =0) |
| **Outcomes** | Signs of adverse drug reactions and infection (defined as sepsis) up to 12 months post surgery |
| **Notes** | Note: only one revision THA patient was included from this paper who was in group 1.  Trial obtained Informed consent and followed guidelines for human experimentation of the Reading Hospital (Reading, PA, USA)  The study received financial assistance from Smith Kline & French Laboratories |

**Risk of bias table**

| **Bias** | **Authors' judgement** | **Support for judgement** |
| --- | --- | --- |
| Random sequence generation (selection bias) |  | Comment: method of randomization not reported |
| Allocation concealment (selection bias) |  | Comment: allocation concealment not reported |
| Blinding of participants and personnel (performance bias) |  | Quote*: "We conducted a double blind study that compared cefonicid to cefazolin in patients undergoing total hip or knee replacement."* Comment: participants and clinicians were blinded to the treatment arms (double-blind study) |
| Blinding of outcome assessment (detection bias) |  | Comment: not clear from the trial report whether the outcome assessor was blinded to treatment allocation |
| Incomplete outcome data (attrition bias) |  | Comment: 3/76 participants that entered the study were not reported on in the results section (1 participant in the cefazolin group and 2 in the cefonicid group), but the reason for this was not clear. However, it was assumed that the one patient identified for this analysis was reported on. |
| Selective reporting (reporting bias) |  | Comment: adverse drug reactions mentioned in methods section but not reported on in the results section. However, infection was reported on - although it was not mentioned in methods section |
| Other bias |  | Comment: the study received financial assistance from Smith Kline & French Laboratories, the manufacturer of the cefonicid |

**Jacobson 2005**

| **Methods** | Single-center RCT, conducted from July 2000-July 2002 |
| --- | --- |
| **Participants** | Participants enrolled in the trial received a primary or revision THA or TKA  Group 1 (revision THA; n = 6): average age = 66.3 years (SD 10.7)  Group 2 (revision THA; n = 3): average age = 64.3 years (SD 13.7)  Groups 1 and 2: male:female = 58%/42%;  Group 3 (revision TKA; n = 6): average age = 68.2 years (SD 10.6)  Group 4 (revision TKA; n = 5): average age = 70.0 years (SD11.2)  Groups 3 and 4: male:female = 40%/60%;  Exclusion criteria: known allergy or hypersensitivity to iodine, acrylate adhesives or adhesive tape; need for bilateral TKA procedures; any infection at time of surgery; previous enrolment in the study, or participation in another study involving an investigational medication within 30 days of enrolment in this study |
| **Interventions** | Group 1 (revision THA; n = 6): preoperative preparation with DuraPrep^TM^ solution plus Ioban^TM^ antimicrobial incise drapes along with systemic IV antimicrobial prophylaxis with majority receiving cefazolin,  Group 2 (revision THA; n = 3): preoperative preparation with povidone iodine plus Ioban antimicrobial incise drapes along with systemic IV antimicrobial prophylaxis with majority receiving cefazolin  Group 3 (revision TKA; n = 6): preoperative preparation with DuraPrep solution plus Ioban antimicrobial incise drapes along with systemic IV antimicrobial prophylaxis with majority receiving cefazolin  Group 4 (revision TKA; n = 5): preoperative preparation with povidone iodine plus Ioban antimicrobial incise drapes along with systemic IV antimicrobial prophylaxis with all receiving cefazolin |
| **Outcomes** | Primary: wound contamination identified by wound culture obtained at the wound edges just prior to wound closure  Secondary: drape lift during the procedure (an indicator that drape adhesion was maintained and thus a sterile surface was maintained throughout the procedure); costs of skin preparation, and 30 day postoperative wound complications (including SSI) |
| **Notes** | Study conducted at the Mayo Clinic, Rochester, MN, USA  IRB approval was obtained and all participants gave written consent  No infections at 30 days were noted in any of the groups |

**Risk of bias table**

| **Bias** | **Authors' judgement** | **Support for judgement** |
| --- | --- | --- |
| Random sequence generation (selection bias) |  | Quote from corresponding author, Doug Osmon, MD, via email dated 5 June 2014, *"The contract statistician developed the randomization scheme using SAS. The randomization scheme was administered using sealed envelopes."* |
| Allocation concealment (selection bias) |  | Comment: allocation concealment not reported |
| Blinding of participants and personnel (performance bias) |  | Quote from corresponding author, Doug Osmon, MD, *"The patients were blinded to the treatment arms. The physician's assistant (PA), who conducted the randomization in the operating room, prepped and draped the patient prior to the procedure starting."* |
| Blinding of outcome assessment (detection bias) |  | Comment: not clear whether those assessing for cost and wound healing complications were aware of the treatment arm to which participants were allocated. For drape lift, the assessors (PA) of this outcome were aware of the treatment arm to which participants had been allocated. For wound contamination, quote: *"Members of the microbiology laboratory staff were blinded to treatment."* |
| Incomplete outcome data (attrition bias) |  | Comment: according to data obtained from the corresponding author, Doug Osmon, MD, all participants entered into the trial were reported on for the outcomes of wound contamination, adverse events (including infection) and drape lift. However only 42 participants in total were evaluated for cost of skin preparation |
| Selective reporting (reporting bias) |  | Comment: outcomes mentioned in the methods section were reported on in the results section. However, a trial protocol was not obtained |
| Other bias |  | Quote from the corresponding author, Doug Osmon, MD, *"3M (the manufacturer of DuraPrep^TM^ and Ioban^TM^) provided all the funding for the study as well was participated in the study design process, study monitoring, and manuscript writing."* Furthermore, trial report stated: *"One of the authors (Doug Osmon) received funding from 3M company through the Mayo/3M Infection Control Alliance."* |

**Mauerhan 1994**

| **Methods** | Multicenter (15 centers) double-blind RCT, conducted from November 1989-April 1991 |
| --- | --- |
| **Participants** | Patients receiving either revision THA or TKA. There were 131 patients receiving a revision THA and 62 receiving a revision TKA. Patients receiving either primary THA or TKA or a revision (second) hip or knee implant (primary implants were excluded from this review and made up 546 hip and 615 knee implants)  Exclusion criteria: allergy to cephalosporins, renal impairment (a serum creatinine level of more than 229 micromoles/l), and neutropenia (< 1000 granulocytes/mm^3^)  Age could not be broken out by revision procedures. However, the average age for both groups combined = 65; male:female = 533:821 (primary and revision procedures) |
| **Interventions** | Group 1: cefuroxime (2nd generation cephalosporin) 1.5 g IV administered 15-60 min prior to surgery followed by 750 mg at 8 h and 16 h after surgery (62 for hip and 29 for knee revision)  Group 2: cefazolin (1st generation cephalosporin) 1 g IV administered 15-60 min prior to surgery and every 8 h for 9 doses (3 days total) (69 for hip and 33 for knee revisions) |
| **Outcomes** | Classified wound infections as superficial or deep, depending upon whether they had developed above or below the fascia.  Deep wound infection, identified from culture specimens of any purulent drainage from inflamed wound, assessed up to 1 year post procedure.  Drug-related adverse events. |
| **Notes** | Study conducted in the USA  A power calculation was performed at a 5% difference in the over-all rate of infection between the 2 groups in the evaluable population at a power of 85%  An intention-to-treat analysis was performed  Protocol approved by the institutional review board for each center and written informed consent was obtained  For revision THA, there was one deep and zero superficial infections in group 1 (cefuroxime) and one deep and one superficial infections in group 2 (cefazolin) [Note: only included those patients who did not experience a major protocol violation.]  For revision TKA, there were zero deep and superficial infections in group 1 (cefuroxime) and zero deep and one superficial infection in group 2 (cefazolin) [Note: only included those patients who did not experience a major protocol violation.]  Unclear as to why revisions occurred. |

**Risk of bias table**

| **Bias** | **Authors' judgement** | **Support for judgement** |
| --- | --- | --- |
| Random sequence generation (selection bias) |  | Quote: *"Randomization schedule was generated by computer for each investigator before study and was administered on the basis of the chronological order in which their patients had been in the study."* |
| Allocation concealment (selection bias) |  | Comment: allocation concealment not reported |
| Blinding of participants and personnel (performance bias) |  | Quote: *"Eligible patients were stratified according to the operative procedure and then were randomly assigned in a double blind fashion . . . "*  Comment: assumed that both participants and clinicians were blinded to the antibiotic treatment arm |
| Blinding of outcome assessment (detection bias) |  | Comment: not clear whether outcome assessors were blinded |
| Incomplete outcome data (attrition bias) |  | Comment: 520/1354 participants (38%; 259 in Group 1and 261 in Group 2) were excluded due to having at least 1 major protocol violation, such as administration of additional antibiotics during the perioperative period or incorrect timing of the first dose. This left 834 participants who were evaluated.  The analysis completed for the outcomes listed above excluded the major protocol violations. |
| Selective reporting (reporting bias) |  | Comment: reported on infection as outlined in the methods section. However, a trial protocol was not obtained |
| Other bias |  | Comment: identified no other biases |

**Phillips 2014**

| **Methods** | Single-center RCT, conducted from March 2011-March 2012 |
| --- | --- |
| **Participants** | People undergoing either primary or revision THA (n = 591) or TKA (n = 596) or spine fusion.  Demographics of primary THA group:   - Group 1 (n = 298): median age = 63 years (21-89); male:female = 123/175; - Group 2 (n = 293); median age = 63 years (30-84); male:female = 120/173   Demographics of primary TKA group:   - Group 1 (n = 299); median age = 63 years (36-93); male:female = 100/199; - Group 2 (n = 297); median age = 63 years (41-92); male:female = 102/195   Exclusion criteria: pregnancy, breastfeeding, allergy to mupirocin or povidone-iodine, interval from pre-surgical assessment clinic visit to surgery of less than 7 days and an infectious indication for surgery. The need for nasal intubation was added as an exclusion criterion shortly after study initiation (typically for cervical spine surgery)  ***Note: In group 1, there were 35 revision THAs and 24 revision TKAs. In group 2, there were 29 revision THAs, and 24 revision TKAs which was the population used in this analysis.*** |
| **Interventions** | Group 1: twice daily application of nasal mupirocin ointment (Bactroban, Nasal®, mupirocin calcium ointment 2%, GlaxoSmithKline) on the intranasal mucosal surfaces of each nostril for 5 days prior to surgery (THA = 298 participants; TKA = 299 participants)  Group 2: 2 applications of povidone-iodine solution (3M^TM^ Skin and Nasal Antiseptic, povidone-iodine solution 5% w/w, 3M Corporation) on the intranasal mucosal surfaces of each nostril (using a cotton swab) within 2 h of surgical incision (THA = 293; TKA = 297)  Both groups also received: 6 chlorhexidine wipes (2% Chlorhexidine Gluconate Cloth Patient Preoperative Skin Preparation, Sage Products) used on the skin the evening before and again on the morning of surgery. 1 wipe was used for each of the following 6 skin areas: neck/chest/arms, abdomen/groin, right leg/foot, left leg/foot, back and buttocks. At the time of surgery all participants received routine antimicrobial prophylaxis which consisted of cefazolin 1 g or clindamycin 600 mg (if participant reported a ß-lactum allergy or vancomycin 1 g for those colonized with MRSA. Antibiotic infusion was started within 1 h of incision or 2 h for vancomycin and additional doses given during surgery according to accepted guidelines. Standard pre-operative surgical site skin preparation also consisted of 2% chlorhexidine gluconate/70% isopropyl alcohol solution |
| **Outcomes** | Deep surgical site infection within 3 months after surgery according to CDC definition  Adverse events related to mupirocin and povidone-iodine application that included: headache, rhinorrhoea (nasal cavity filling with fluid), nasal irritation, congestion, cough, and pharyngeal irritation |
| **Notes** | Trial conducted at New York University  Trial was registered on ClinicalTrials.gov website under clinical trial number:NCT01313182  3M Corporation provided a research grant for the study  Preoperative *S aureus* antibiotic susceptibility testing and strain typing was performed including testing for MRSA  Reached out repeatedly by email to Michael Phillips, principal investigator and lead author on: 25 August, 27 August, 1 Sept, and 3 Sept. Was ultimately told by Michael Phillips that he did not have the time to separate out the infection incidence in revision procedures.  Based on prior correspondence with Michael Phillips on 6 March 2014 was able to determine that the incidence of infections (using the modified intent to treat criteria [MITT]) in primary TKA for mupirocin was 8 out of 299 procedures performed and for primary THA 0 out of 298 procedures performed. Further the incidence of infections for MITT in primary TKA for the povidone iodine (PI) group was 3 out of 297 procedures performed and for primary THA 1 out 293 procedures performed. Thus in the remaining procedures performed (for spine fusion, spine fusion revision, knee revision, hip revision, shoulder, and shoulder revision) there were 258 procedures in the mupirocin group in which 6 additional infections occurred (incidence of 6/258 = 2.3%) and; 252 procedures in the PI group in which 3 infections occurred (3/252 = 1.2%). As well, since there were 24 revision TKA procedures and 35 revision THA procedures performed in the mupirocin group and 24 revision TKA and 29 revision THA procedures in the PI group; this incidence of infection was apportioned (estimated to be) for each treatment group - e.g. for mupirocin revision TKA and THA groups the incidence of infection = 2.3% and; for PI revision TKA and THA groups the incidence of infection = 1.2%. This further translated into for the mupirocin group: 1 infection in revision THA and 1 infection in revision TKA and for the PI group: 0 infections in the revision THA group and 0 infections in the revision TKA group. In a sensitivity analysis and examining the worst case scenario, apportioning all 6 infections to either mupirocin revision TKA or revision THA and 0 infections to either PI revision TKA or THA resulting in non-significantly different findings. |

**Risk of bias table**

| **Bias** | **Authors' judgement** | **Support for judgement** |
| --- | --- | --- |
| Random sequence generation (selection bias) |  | Quote: *"Subjects were stratified by arthroplasty or spine fusion surgery, and then randomized 50:50 to either mupirocin or povidone-iodine treatment groups in blocks of 100."* |
| Allocation concealment (selection bias) |  | Comment: allocation concealment not reported |
| Blinding of participants and personnel (performance bias) |  | Comment: physicians and participants knew the group to which they had been allocated |
| Blinding of outcome assessment (detection bias) |  | Quote: *"Infection Prevention and Control practitioners reviewing the records were blinded to study participation and receipt of study treatment."* |
| Incomplete outcome data (attrition bias) |  | Comment: all participants were reported on via an intention-to-treat analysis |
| Selective reporting (reporting bias) |  | Comment: protocol defined the following outcomes: deep and superficial infection at 12 months; length of hospital stay due to re-admission; readmission within 12 months of the procedure; adverse events to mupirocin and povidone-iodine. The study evaluated participants at 3 months only for infection and adverse events |
| Other bias |  | Comment: 3M Corporation provided a research grant for this study |

**Excluded study – characteristics**

**Adalberth 2002**

| **Reason for exclusion** | RCT of primary TKA only |
| --- | --- |

**Bohm 2012**

| **Reason for exclusion** | RCT of primary THA only |
| --- | --- |

**Bryan 1988**

| **Reason for exclusion** | RCT of cefazolin versus cefamandole in total joint arthroplasty - primary hip, primary knee, and revision procedures. However, unable to separate the infections reported by procedure type. An attempt was made to contact the lead author, Charles S Bryan MD, to obtain this information. An email was sent to[cbryan@gw.mp.sc.edu](mailto:cbryan@gw.mp.sc.edu) on 23 August 2015, but bounced back as undeliverable. |
| --- | --- |

**Byren 2012**

| **Reason for exclusion** | Excluded due to use of daptomycin for treatment of infection in revision arthroplasty vs. use in antibiotic prophylaxis |
| --- | --- |

**Chareancholvanich 2012**

| **Reason for exclusion** | RCT of primary TKA only |
| --- | --- |

**Chiu 2001**

| **Reason for exclusion** | QRCT with randomization performed on an odd-even basis according to medical record number. Not a true RCT and only evaluated primary TKA. |
| --- | --- |

**Chiu 2002**

| **Reason for exclusion** | QRCT with randomization performed on an odd-even basis according to medical record number. Not a true RCT and only evaluated primary TKA. |
| --- | --- |

**Davis 1987**

| **Reason for exclusion** | RCT however only performed on patients undergoing primary THA (70) and 39 undergoing primary TKA |
| --- | --- |

**De Lalla 1993**

| **Reason for exclusion** | RCT - excluded as it only addressed primary TKA and did not evaluate endpoint of infection |
| --- | --- |

**Ericson 1973**

| **Reason for exclusion** | RCT however only performed on patients undergoing primary THA. Carlsson 1977 follow up study was also excluded for the same reason. |
| --- | --- |

**Evard 1988**

| **Reason for exclusion** | RCT however only examined primary THA. |
| --- | --- |

**Friedman 1990**

| **Reason for exclusion** | RCT however only examined primary TKA and did not examine endpoint of infection. |
| --- | --- |

**Garcia 1991**

| **Reason for exclusion** | RCT however only included the Moore endoprosthesis, which is not a total joing replacement |
| --- | --- |

**Gilliam 1990**

| **Reason for exclusion** | RCT comparing preoperative skin preparation using a 5 minute aqueous iodophor scrub followed by application of aqueous iodophor solution as a paint versus skin prepared with a 1-step application of water-insoluble iodophor in alcohol solution applied as a paint. 60 participants randomized. Not possible to determine if the total joint surgeries were primary or revision in nature and whether they were total knee or hip |
| --- | --- |

**Gunst 1984**

| **Reason for exclusion** | RCT - however performed only on primary THA patients |
| --- | --- |

**Hill 1981**

| **Reason for exclusion** | RCT - however performed only on primary THA patients |
| --- | --- |

**Hinarejos 2013**

| **Reason for exclusion** | RCT of primary TKA implants only |
| --- | --- |

**Johnson 1987**

| **Reason for exclusion** | RCT - however did not examine the endpoint of infection and was performed only on primary TKA patients. |
| --- | --- |

**Jones 1987**

| **Reason for exclusion** | RCT of various types of surgery comparing timentin to cefoxatime. 25 total joint patients randomized. Excluded because impossible to separate the infections and costs by procedure type (also unclear whether primary total joint implants were knee or hip) |
| --- | --- |

**Jones 1988**

| **Reason for exclusion** | RCT investigating cefoxatime versus other cephalosporins and ticarcillin/clavulanic acid in various surgical procedures, including total joint arthroplasty (320 participants). Excluded because impossible to separate out infections by procedure type. Jones 1987 (American Journal of Surgery) also excluded for the same reason. |
| --- | --- |

**Josefsson 1981**

| **Reason for exclusion** | RCT - however performed on primary total hip arthroplasty patients only and followed up on over a period of 10 years. Josefsson 1990 and Josefsson 1993 also excluded for the same reasons. |
| --- | --- |

**Kanellakopoulou 2009**

| **Reason for exclusion** | RCT of primary THA and TKA. Exclusion criteria specifically mentioned in the study were revision arthroplasty procedures |
| --- | --- |

**Lidwell 1984**

| **Reason for exclusion** | Could not breakout revision procedures from primary THA/TKA |
| --- | --- |

**Liebergall 1995**

| **Reason for exclusion** | RCT which was excluded because it was impossible to separate out infections by procedure type. |
| --- | --- |

**McQueen 1990**

| **Reason for exclusion** | RCT of primary THA and TKA - McQueen 1987 early results and McQueen 1990 (2 year follow up) |
| --- | --- |

**Mollan 1992**

| **Reason for exclusion** | RCT of teicoplanin versus cefamandole. Excluded because impossible to separate out infections by procedure type. Also only primary THA and TKA performed. |
| --- | --- |

**Morrison 2014**

| **Reason for exclusion** | RCT of primary THA and TKA only |
| --- | --- |

**Nelson 1983**

| **Reason for exclusion** | Study stated it was randomized, but a randomization scheme was not used, and participants were divided into 2 groups, according to the last digit (even or odd) of their hospital number. Further, could not break out which patients received primary versus revision THA and TKA. |
| --- | --- |

**Nelson 1993**

| **Reason for exclusion** | RCT of treatment for infection in revision THA and TKA, not for prevention of infection. |
| --- | --- |

**Periti 1994**

| **Reason for exclusion** | RCT; unclear how many people received a hip or knee implant. Excluded because impossible to separate infections by procedure type |
| --- | --- |

**Periti 1999**

| **Reason for exclusion** | RCT; excluded because impossible to separate infections by procedure type. Also only primary THA and TKA performed. |
| --- | --- |

**Pollard 1979**

| **Reason for exclusion** | RCT excluded because vast majority of patients 279 out 297 were primary THA. There were 4 procedures performed on failed THA (i.e. revision). However, of these 4, it could not be determined who received flucloxacillin or cephaloridine prophylaxis. |
| --- | --- |

**Richardson 1993**

| **Reason for exclusion** | RCT excluded because all were primary TKA procedures and did not address the end point of infection. |
| --- | --- |

**Ritter 1989**

| **Reason for exclusion** | RCT; excluded due to only primary THA and TKA procedures being performed |
| --- | --- |

**Schulitz 1980**

| **Reason for exclusion** | Study specifically excluded previous operations at the site of surgery. |
| --- | --- |

**Soave 1986**

| **Reason for exclusion** | RCT; excluded because it could not be determined whether patients had received a primary or revision hip or knee. it was assumed that the patients had received primary total joint implants |
| --- | --- |

**Soriano 2008**

| **Reason for exclusion** | RCT; excluded due to only primary TKA being performed. |
| --- | --- |

**Suter 1994**

| **Reason for exclusion** | RCT; excluded due to only primary THA being performed. |
| --- | --- |

**Tetreault 2014**

| **Reason for exclusion** | RCT - however, did not evaluate incidence of infection after revision arthroplasty in these patients. Patients entered into trial had known prosthetic joint infection of a THA or TKA. |
| --- | --- |

**Tyllianakis 2010**

| **Reason for exclusion** | RCT; excluded due to only primary TKA and THA performed. |
| --- | --- |

**Vainionpää 1988**

| **Reason for exclusion** | RCT of primary THA and TKA only. As well, did not measure the endpoint of infection. Endpoint in study was concentration levels of antibiotics in the synovial fluid of the patients. |
| --- | --- |

**van den Brand 2001**

| **Reason for exclusion** | RCT. However only evaluated primary THA and TKA. |
| --- | --- |

**van Kasteren 2007**

| **Reason for exclusion** | Retrospective review of timing of antibiotic prophylaxis |
| --- | --- |

**Van Meir haaeghe 1989**

| **Reason for exclusion** | Could not break out revisions from primary THA/TKA |
| --- | --- |

**van Rijen 2012**

| **Reason for exclusion** | RCT of primary THA and TKA only. |
| --- | --- |

**Wall 1988**

| **Reason for exclusion** | RCT excluded because implant was an endoprosthetic hip or knee (i.e. only one portion of the joint was replaced) and was not a total hip or knee replacement |
| --- | --- |

**Winter 1987**

| **Reason for exclusion** | RCT, however, it was not clear whether the type of antibiotic prophylaxis administered was determined by the type of procedure (hip or knee). Also not clear whether implant was a total knee or hip implant as implants were identified as endo-prostheses. |
| --- | --- |

**Young 2012**

| **Reason for exclusion** | RCT of use of intraosseous regional administration (IORA) with cefazolin in patients undergoing TKA |
| --- | --- |

**Young 2014**

| **Reason for exclusion** | RCT of use of intraosseous regional administration (IORA) with vancomycin in patients undergoing primary TKA. |
| --- | --- |

**Zdeblick 1986**

| **Reason for exclusion** | RCT of elective adult orthopedic surgical cases that excluded total joint arthroplasty patients |
| --- | --- |

**Zimmerli 1998**

| **Reason for exclusion** | RCT of implant infections that were not revised but debrided and treated with IV course of flucloxacilln or vancomycin with rifampin or placebo. |
| --- | --- |

**Characteristics of ongoing studies**

**NCT01175044**

| **Methods** | RCT single center trial undertaken from August 2010 to the present |
| --- | --- |
| **Participants** | Patients receiving a revision TKA. Exclusion criteria included:   - Age ≤17 years - Allergy to povidone iodine - Any condition requiring antibiotics 14 days prior to arriving for surgery - Chronic immunosuppression (e.g. HIV/AIDS) - Unable to adhere to follow up schedule and treatment - Patients scheduled to undergo revision TKA for infectious reasons - Inability to provide informed consent or to comply with study assessments |
| **Interventions** | Revision TKA with a dilute betadine lavage prior to surgical closure for 3 minutes followed by 2000 ml of sterile saline irrigation  Revision TKA with 2000 ml sterile saline lavage only.  All other treatments will be standard of care and the same in both groups. |
| **Outcomes** | Infection rate at 12 month follow up. |
| **Notes** | Study performed at Rush University Medical Center, Chicago, IL  Contacted principal investigator on 9/5/15 seeking an update as to when the trial would be completed. Anticipated enrollment of 600 revision TKAs. Reply on 9/5/15 from Craig Della Valle was that they are still enrolling patients and will not have the results for some time. |

**NCT02020031**

| **Methods** | RCT; single center trial being undertaken from December 2013 to December 2015 |
| --- | --- |
| **Participants** | Patients receiving revision TKA. Exclusion criteria include:   - Current treatment with IV vancomycin within the preceding 7 days - Previous hypersensitivity to vancomycin - Significant cardiac or respiratory abnormality - Contraindications to intraosseous vascular access using the EZ-IO (from Vidacare) - Sepsis |
| **Interventions** | Group 1: 500mg vancomycin intraosseous administration into the proximal tibial cannula, after tourniquet inflation and immediately prior to skin incision) plus IV cefazolin prior to the beginning of surgery and; 3 postoperative dosed of cefazolin over a 24-hour period.  Group 2: 1g IV vancomycin administered via forearm vein, given over a one-hour infusion timed to finish approximately 30 minutes prior to tourniquet inflation plus IV cefazolin prior to the beginning of surgery and; 3 postoperative dosed of cefazolin over a 24-hour period. |
| **Outcomes** | Compare average level of concentration between the 2 groups [baseline to 24 yours post procedure] |
| **Notes** | Study performed at Mayo Clinic  Contacted research coordinator on 8/25 and 9/11 (Debra Ryan) via email. On 9/16, principal investigator, Mark Spangehl, MD, replied that the study would not be completed until December 2015. |

**NCT02469311**

| **Methods** | RCT; single center trial undertaken from March 2012 to October 2015 |
| --- | --- |
| **Participants** | Patients receiving primary or revision TKA or THA. Exclusion criteria included:   - Pregnant - <18 years of age - Chronic hepatitis B or C infection - Infection in or around the joint requiring surgery - >10 milligrams of prednisone equivalent for >10 days with 90 days prior to enrollment - History of immunosuppresive disease - Unable to comply with study requirements |
| **Interventions** | Revision TKA/THA with use of chlorhexidine cloth the night before and the morning prior to surgery (prior to admission and at home).  Revision TKA/THA with standard of care bathing with antibacterial soap and water the night before surgery (prior to admission and at home).  Both groups received standard infection control practices during admission |
| **Outcomes** | Periprosthetic infection at one year and incidence of adverse events |
| **Notes** | Preliminary results showed that the use of cloths helped prevent infections.  On 9/5/15, Dr. Michael Mont, principal investigator and Director of the Rubin Institute for Advanced Orthopedics and Joint Preservation and Replacement at LifeBridge Health, was contacted via email requesting data on the infection incidence seen in revision THA/TKA. |
